# Identification of genes associated with longevity in dogs: 9 candidate genes described in Cavalier King Charles Spaniel

**DOI:** 10.1016/j.vas.2024.100420

**Published:** 2024-12-16

**Authors:** Evžen Korec, Lenka Ungrová, Josef Kalvas, Jiří Hejnar

**Affiliations:** a ZOO Tábor a.s., Dukelských Hrdinů 19, 170 00 Prague 7, Czech Republic; b Institute of Molecular Genetics of the Czech Academy of Sciences, Vídeňská 1083, 142 20 Prague 4, Czech Republic

**Keywords:** Longevity-associated genes, GWAS, Cavalier King Charles Spaniel dog, Extending lifespan, Longevity testing

## Abstract

In the past years, dogs have served as a convenient natural model organism for longevity due to their similarity with humans concerning not only their environment but also the diseases and complications occurring in older age. Since many dog breeds have significantly shorter lifespan than their closely related breeds, identification of genes associated with longevity may help to elucidate its background and serve as a possible tool for selective breeding of long-living dogs. This genome-wide association study (GWAS) was undertaken to identify the candidate genes associated with longevity in Cavalier King Charles Spaniel individuals that have reached the age of more than 13 years. We described 15 SNPs localized in nine genes: *B3GALNT1, NLRP1* like, *PARP14, IQCJ-SCHIP1, COL9A1, COL19A1, SDHAF4, B3GAT2,* and *DIRC2* that are associated with longevity in purebred Cavalier King Charles Spaniels. These results are promising for future research and possible selective breeding of companion dogs with extended lifespan.

## 1 Introduction

Understanding the genetic factors underlying longevity in vertebrates, including humans and dogs, has been a topic of growing interest in the past decades (Brooks-Wilson, 2013; Korec et al., 2022a; Mayne et al., 2019; Singh et al., 2019).

In the past years, dogs have become an interesting natural model organism for studying a variety of genetic factors, including human diseases and longevity. Dogs and humans have many diseases in common, resembling in symptoms, frequency, and genetic factors that are involved in the disease development. They also share the same environment that can influence the disease onset or its progression (Sherain et al., 2010; Switonski, 2014). Selective breeding, interbreed diversity, low genetic diversity within the breeds, and similar quality of healthcare compared to humans make dogs a suitable natural model organism for studying genetic background and other factors underlying aging and longevity (Gilmore et al., 2015; Hoffman et al., 2018).

With growing interest in aging and longevity, the focus has shifted toward not only the life expectancy extension but also to the healthspan— the length of life spent in good health, free from chronic disease or disability. Understanding the factors that promote healthy longevity in humans requires insights from suitable models, and dogs provide a unique opportunity to investigate these processes due to their similarities with humans (Gilmore et al., 2015; Hoffman et al., 2018). There is an important difference between humans and dogs considering the definition of lifespan and healthspan. In dogs, these two terms represent similar period of time. After the development of chronic diseases and disabilities in older aged dogs, owners usually resort to euthanasia because the trade-off between the quality and quantity of life is hard to assess (Mondino et al., 2024).

Since dogs are the most favourite human companions in the USA and the EU (AVMA, 2022), an extended lifespan associated with good health is also a desirable trait in dog breeding. Nevertheless, the recent knowledge does not allow significant extension of the lifespan by intentional breeding in dogs or animals in general.

The Cavalier King Charles Spaniel (CKCS), classified as a toy dog breed, was bred from the English Toy Spaniel in the early 20th century. The English Toy Spaniel (also known as King Charles Spaniel) originated in England in the 17th century from small Spaniel toy breeds brought from Asia. The breed was recognized by the Fédération Cynologique Internationale (FCI) in 1955 and the American Kennel Club (AKC) in 1995. Even though CKCS is considered a small breed, its median lifespan of 9.9 years (O'Neill et al., 2013) is remarkably comparable to much larger breeds, such as the Cane corso (median lifespan of 9.29 years (Korec et al., 2017)). Large dog breeds typically have shorter lifespan compared to smaller breeds (Kraus et al., 2013), but CKCS represents an exception to this trend. Additionally, median lifespan of CKCS is drastically lower compared to the most closely related breed, the King Charles Spaniel, whose median lifespan is 12.0 years (O'Neill et al., 2013). The most common diseases affecting CKCS are degenerative mitral valve disease of the heart (Swift et al., 2017) and syringomyelia (Knowler et al., 2017; Pedersen et al., 2024). Heart diseases are the cause of death in more than 37 % of dogs (Egenvall et al., 2006) and they are ranked as the main cause of death in the CKCS (Lewis et al., 2018). Taken together, all these facts make the CKCS breed an interesting model for identifying genes associated with dog longevity, as long-living individuals of this breed are likely to have an exceptional genetic predisposition to reach a long age, probably influencing both lifespan and healthspan.

This study was performed as an extension of our previous work (Korec et al., 2022b), with the aim to use the established methodology to identify candidate SNPs and genes associated with longevity in purebred Cavalier King Charles Spaniel (CKCS) dogs using genome-wide association study (GWAS).

## 2 Material and methods

### 2.1 Sample collection and experimental groups

Buccal swab samples from purebred, FCI registered, CKCS dogs were collected during the year 2023 with the help of registered breeders and kennels in the Czech Republic. For the purpose of this research, samples were divided into two groups defined by the age of the dogs examined. For the reference group, we sampled dogs at the age between 2 and 7 years, which present the first quartile in the range of mortality age in the CKCS. The group of long-lived dogs contained samples from individuals older than 13 years, which presents the upper quartile in the range of mortality age in CKCS (O'Neill et al., 2013). Since some dogs from the reference group could be long-living, monitoring of these dogs will continue to confirm our results. The health status of the dogs was not evaluated.

### 2.2 DNA isolation and sample preparation

DNA was isolated from 69 buccal swab samples (Table S1) using a standard phenol-chloroform DNA isolation protocol. The concentration and purity of isolated DNA were checked using a spectrophotometer. The quality and length of the isolated DNA that are required for SNP genotyping were checked on 2 % agarose gel. Overall, 26 samples of long-lived dogs and 22 samples of reference dogs were selected for subsequent analysis. The remaining 21 samples did not pass the DNA quality check and had insufficient quality for SNP genotyping. Samples were genotyped using the Illumina CanineHD Beadchip at Neogen (Ayr, Scotland).

### 2.3 GWAS and statistical analysis

Data clean-up, association analysis and statistical tests of the genotyping data were performed as in Korec et al. (2022b). After initial data clean-up, 92,679 variants and 41 dogs (24 long-lived and 17 reference) were used for the association analysis. Association analysis was performed using 1df chi-square allelic test.

The statistical significance of the distribution of genotypes from all samples within the long-lived and reference groups was tested in R using Fisher's exact test (Fisher, 1934).

### 2.4 Results verification

According to the result of GWAS, the genomic position of all candidate SNPs was checked in the CanFam 3.1 reference genome. Only SNPs that are present in an annotated gene or locus were selected for further analysis.

Human homologous sequences of the genes in which significantly associated SNPs were present were retrieved from the UNIPROT database (Release 2024_01). Gene ontologies for the selected genes were then collected to evaluate the potential effect of the longevity associated SNPs in a broader context of biological processes.

InterproScan v2.1 (Quevillon et al., 2005) in Geneious software (v2023.2.1, https://www.geneious.com) was used to assess the change of amino acids and affected protein domains. The effect of amino acid changes on the structure of the proteins associated with longevity was checked using Alphafold (Jumper et al., 2021).

### 2.5 Data visualisation

Manhattan plot for visualization of the association analysis, PCA plot, and qqplot of the association analysis were constructed in R version 4.3.2 (R Core Team, 2021) using R Studio version 2023.12.0 with packages ggplot2 (Villanueva & Chen, 2019), qqman (Turner, 2018) and lattice (Sarkar, 2008) that are available in the CRAN repository.

Protein structures were visualized in ChimeraX (Meng et al., 2023).

## 3 Results

### 3.1 GWAS

**Fig. 1:**
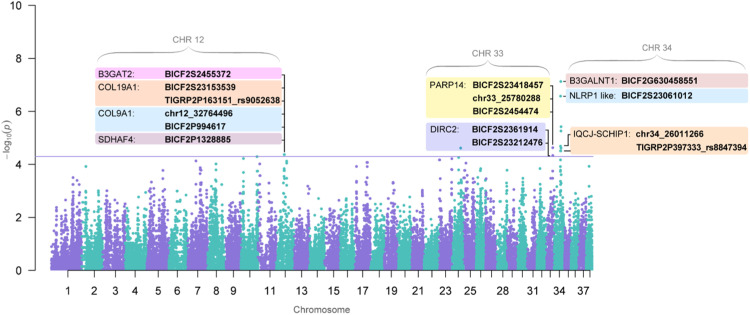
Manhattan plot of the GWAS results. The blue line represents a significance threshold of 5.0e-05.

Out of the 48 genotyped dogs, 41 (24 long-lived and 17 reference) passed the filtering steps. 7 dogs were excluded due to the low genotyping rate (Table S1). The PCA plot and qqplot show little stratification that is due to related animals (Fig. S1, S2). After the association analysis, 31 SNPs passed through the predetermined significance threshold (*P*-value = 5.0e-05). Fifteen SNPs that passed the significance threshold were localized within nine annotated genes in the CanFam 3.1 reference genome (Fig. 1, Table 1). Out of those, 12 SNPs were found in introns, two in exons and one in the 3’UTR region. These 15 SNPs were chosen for further investigation.

**Table 1:** Table of GWAS results for 15 SNPs sorted by the highest P-value. Chr, chromosome; SNP, single nucleotide polymorphism; A1, minor allele; F_A, frequency of minor allele in the long-lived group; F_U, frequency of minor allele in reference group; A2, major allele; CHISQ, basic allelic chi-square test; OR, estimated odds ratio

Gene	*P*-value	Position	Chr	SNP	Base pair	A1	F_A	F_U	A2	CHISQ	OR
B3GALNT1	7,44E-08	Intron	**34**	BICF2G630458551	27100531	A	0,13	0,71	G	28,95	0,06
NLRP1 like	2,66E-07	Intron	**34**	BICF2S23061012	26166584	A	0,13	0,68	G	26,48	0,07
PARP14	2,34E-05	Intron	**33**	BICF2S23418457	25772821	G	0,50	0,06	T	17,89	16,00
PARP14	2,34E-05	Exon	**33**	chr33_25780288	25780288	G	0,50	0,06	A	17,89	16,00
PARP14	2,34E-05	Exon	**33**	BICF2S2454474	25797175	C	0,50	0,06	T	17,89	16,00
IQCJ-SCHIP1	2,06E-05	Intron	**34**	chr34_26011266	26011266	A	0,21	0,68	G	18,14	0,13
IQCJ-SCHIP1	3,10E-05	intron	**34**	TIGRP2P397333_rs8847394	25663741	A	0,04	0,41	G	17,36	0,06
COL9A1	4,26E-05	Exon_3´UTR	**12**	chr12_32764496	32764496	C	0,56	0,12	T	16,75	9,64
COL9A1	4,26E-05	Intron	**12**	BICF2P994617	32846764	C	0,56	0,12	T	16,75	9,64
COL19A1	4,26E-05	Intron	**12**	BICF2S23153539	32620063	T	0,56	0,12	C	16,75	9,64
COL19A1	4,26E-05	Intron	**12**	TIGRP2P163151_rs9052638	32712786	A	0,56	0,12	G	16,75	9,64
SDHAF4	4,26E-05	Intron	**12**	BICF2P1328885	33086265	C	0,56	0,12	T	16,75	9,64
B3GAT2	4,26E-05	Intron	**12**	BICF2S2455372	33347976	G	0,56	0,12	A	16,75	9,64
DIRC2	4,63E-05	Intron	**33**	BICF2S2361914	25875733	A	0,48	0,06	G	16,59	14,72
DIRC2	4,63E-05	Intron	**33**	BICF2S23212476	25881402	G	0,48	0,06	A	16,59	14,72

Two SNPs, BICF2G630458551 in the *B3GALNT1* (beta-1,3-N-acetylgalactosaminyltransferase 1) gene and BICF2S23061012 in the *NLRP1* (NLR family pyrin domain containing 1) like gene (LOC102153616, not annotated yet in dogs), showed a higher association (*P*-value = 7.44e-08 and 2.66e-07 respectively) with longevity in comparison with the rest of the dataset. The association with longevity was also shown for SNPs in *PARP14* (poly (ADP-ribose) polymerase family member 14), *IQCJ-SCHIP1* (IQ motif containing J and schwannomin interacting protein 1), *COL9A1* (collagen type IX alpha 1 chain), *COL19A1* (collagen type XIX alpha 1 chain), *SDHAF4* (succinate dehydrogenase complex assembly factor 4), *B3GAT2* (beta-1,3-glucuronyltransferase 2), and *DIRC2* (disrupted in renal carcinoma 2) genes (Table 1).

**Fig. 2:**
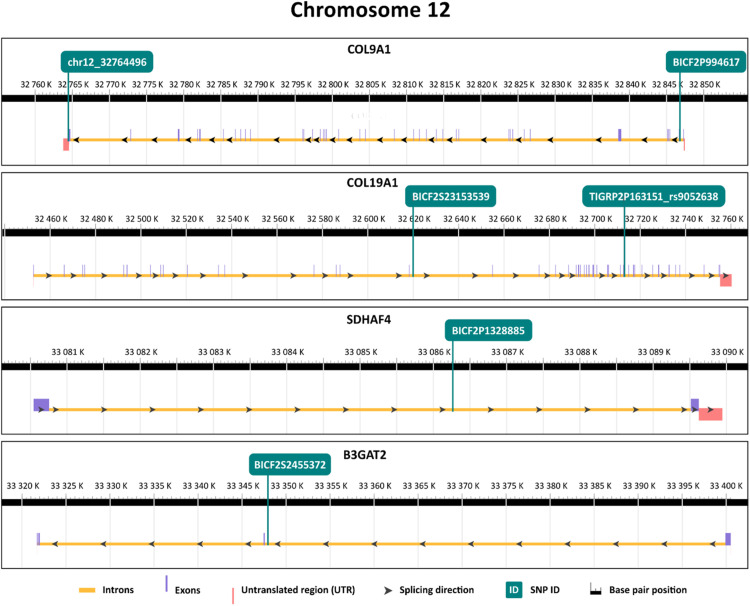

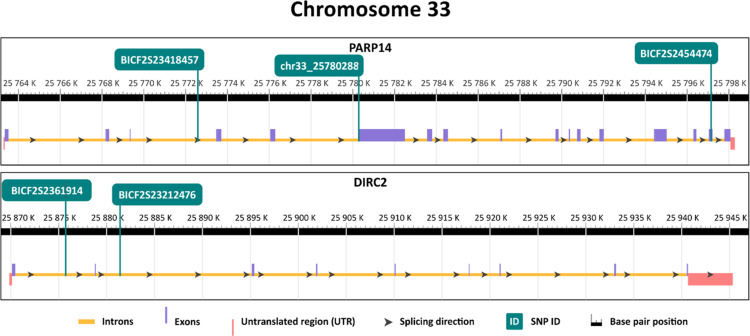

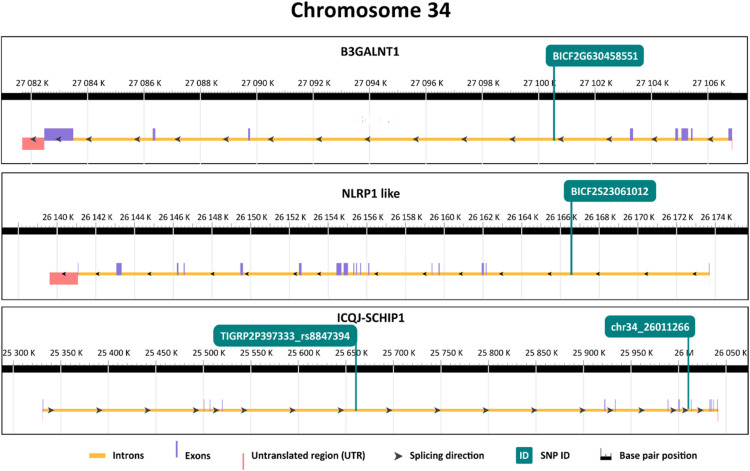
Position of selected SNPs significantly associated with longevity in respective genes. The yellow line represents introns, purple line represents exons and the red line in the scheme represents UTRs. Each position of a particular SNP in the respective genes is visualised by the green line that is annotated at the top.

These genes are located on chromosome 34 (*B3GALNT1, NLRP1* like, *IQCJ-SCHIP1*) chromosome 33 (*PARP14, DIRC2*) and chromosome 12 (*COL9A1, COL19A1, SDHAF4, B3GAT2*) (Table 1). In the *PARP14, IQCJ-SCHIP1, COL9A1, COL19A1*, and *DIRC2* genes, more linked SNPs showing association with longevity were found. The exact positions of SNPs in their respective genes are displayed in Fig. 2.

### 3.2 Longevity-associated genotypes

**Fig. 3:**
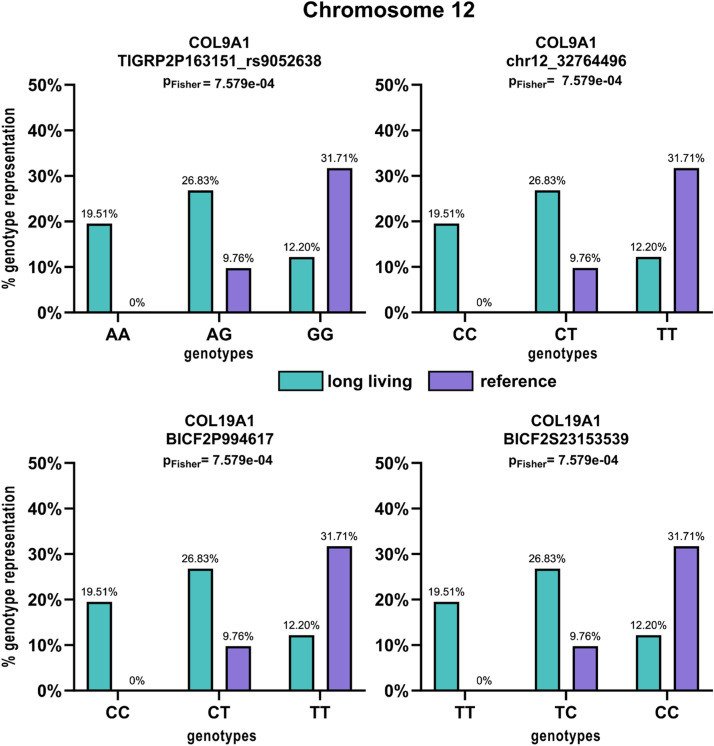

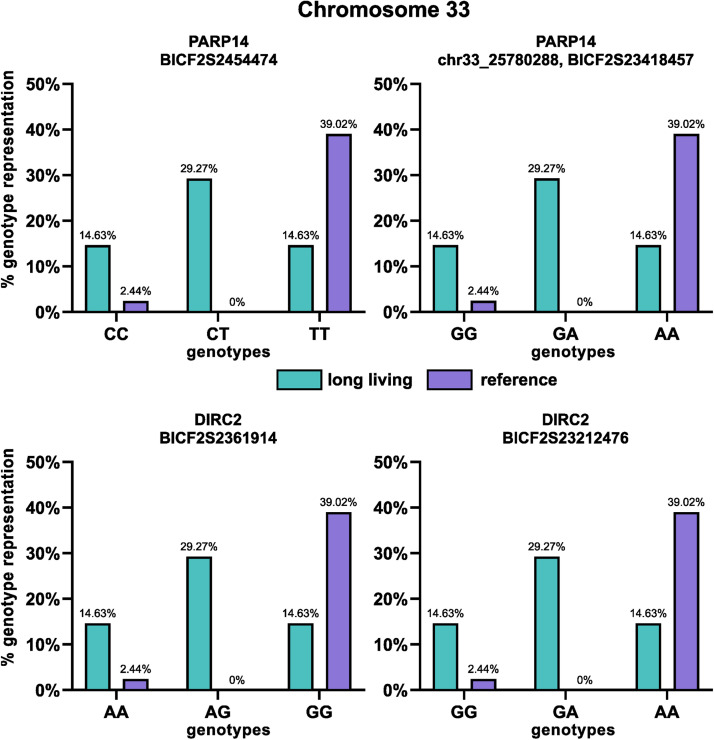

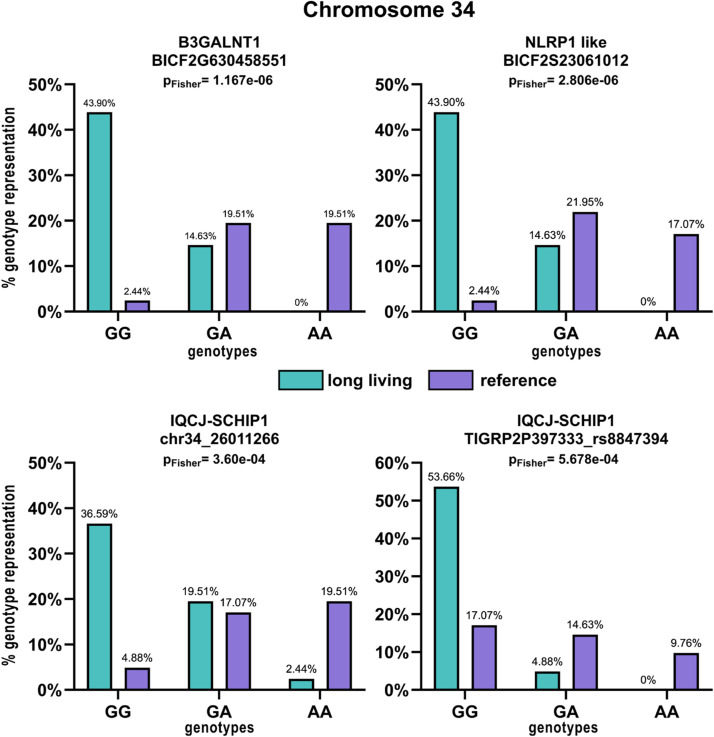
Distribution of genotypes in the significant SNPs associated with longevity in the investigated genes. Turquoise colour represents the long-living group and purple colour represents the reference group. Each graph contains the P-value from Fisher's exact test.

The genotypes in all the SNPs’ positions in the studied dogs were retrieved from PLINK raw files and the differences between the long-lived and reference groups are visualized using a column plot (Fig. 3) together with the significance values from the Fisher's exact test.

Both, the most significant SNPs (BICF2G630458551 in the *B3GALTN1* gene and BICF2S23061012 in the *NLRP1* like gene) show significant association of the long-living group with the GG homozygous and GA heterozygous genotypes compared to the reference group (*P*-value = 1.167e-06 and 2.806e-06 respectively).

The distribution of particular genotypes of SNPs in particular genes and P-values from the Fisher's exact test are shown in Fig. 3. All longevity-associated SNPs on chromosome 12 and 33 were found to be linked with the same genotype distributions.

The SNP chr33_25780288 in *PARP14* was the only SNP that would lead to a change of an amino acid in the protein sequence. Long-living individuals show an amino acid change from methionine (ATG) to valine (GTG) (Fig. S3). According to Alphafold predictions, this change does not have any significant effect on the structure of the protein (Fig. S4).

Association with longevity of the G allele as well as the homozygous and heterozygous genotype GG/GA was significant in the Fisher's exact test (*P*-value = 2.289e-05 and 1.039e-05). The nucleotide change (T->C) in BICF252454474 in an exon of the *PARP14* gene does not cause an amino acid change.

## 4 Discussion

In this study, we describe SNPs in nine genes that are associated with longevity in the Cavalier King Charles Spaniel dog breed. We used GWAS as a tool for selecting the candidate SNPs.

According to GWAS, the most significant SNP associated with longevity was located in *B3GALNT1* gene. The *B3GALNT1* gene encodes beta-1,3-galactosyltransferase, an enzyme involved in the synthesis of complex glycoproteins and glycolipids (Honke, 2014). It plays an important role in glycosylation, which is crucial for various cellular processes, including cell adhesion, signalling, and immune response. Changes in glycosylation have been implicated in age-related diseases, including neurodegenerative disorders and cancer, suggesting a potential role in the aging process in humans (Paton et al., 2021). In the past, numerous studies suggested a relationship between galactosyltransferase and cancer, including the use of galactosyltransferase as a cancer biomarker (Weiser et al., 1976; Nozawa et al., 1990; Saitoh et al., 2003). Overall, *B3GALNT1* can play an important role in prolonging life, especially regarding cancer development, but more studies are needed to understand its role in longevity

The second most significant SNP is located in the intron of *NLRP1* (also known as *NALP1*) like gene, which plays an important role in the immune system. It forms intracellular protein complexes called inflammasomes that are involved in most aging-related complications in humans (Barry et al., 2023; Pandey et al., 2021). *NLRP1* is a DNA damage sensor, and it is a major mediator of senescence in aging cells (Muela-Zarzuela et al., 2024). It was also previously found to be associated with longevity in humans (Flachsbart et al., 2010). Altogether, these findings in humans suggest that even in dogs, *NLRP1* can play a crucial role in senescence and aging-related diseases.

Three linked SNPs, associated with longevity, were found in *PARP14*. Two of them are located in an exon with one SNP resulting in amino acid change that does not have any significant effect on the protein structure. However, an alternative start codon is introduced by the mutation. *PARP14* is involved in signalling pathways with relevance to cancer, inflammation, DNA repair, gene transcription, and cell death in humans (Torretta et al., 2023). *PARP14* could indirectly influence longevity by safeguarding genomic integrity and promoting overall cellular health. *PARP14* together with *PARP9* are also associated with inflammation and human coronary artery disease (Iwata et al., 2016). *PARP9* was previously found to be associated with longevity in the Cane corso (Korec et al., 2022b). These results suggest a possible role of the *PARP*s family in longevity since importance of another member of the family, *PARP1*, was also previously investigated in relation to human aging and its role in longevity was also experimentally proven in mouse and fruit flies (Mangerich & Bürkle, 2012; Guo et al., 2023). Importantly, *PARP* genes are also targeted by their inhibitors in treatment of multiple cancers in humans (Lord & Ashworth, 2017) and their use is starting to be also evaluated in dogs (Pawlak et al., 2023; Thumsen-Henner et al.,2020).

Another linked gene associated with longevity found in the current study, *DIRC2*, is located in close proximity to both *PARP14* and *PARP9*. In humans, the primary association of *DIRC2* is with renal carcinoma (Bodmer et al., 2002) but the lysosomal function, in which *DIRC2* plays a crucial role, is vital for cellular homeostasis and aging processes (Carmona-Gutierrez et al., 2016). Age-related downregulation of *DIRC2* expression was also described in multiple mouse and human tissues (Coler-Reilly et al.,2024) but more research is needed to assess its influence on longevity and healthy aging.

Two longevity-associated SNPs were found in an intron of *IQCJ-SCHIP1* gene. *IQCJ-SCHIP1* is a fusion transcript expressed in the brain and was not previously described in the context of longevity (Kwaśnicka-Crawford et al., 2006).

*COL9A1* on chromosome 12, in which two linked SNPs associated with longevity were located, encodes one of the alpha chains of type IX collagen, a protein involved in the structure and function of cartilage. It is also linked with the development of hip osteoarthritis in humans (Bateman, 2005). *COL19A1*, also with two longevity-associated SNPs, encodes a protein belonging to the collagen family involved in forming the extracellular matrix. Moreover, *COL19A1* is considered to be a potential genetic biomarker of longevity in mice with Amyotrophic Lateral Sclerosis (Calvo et al., 2012). Overall, collagens play a role in longevity by maintaining the tissue integrity and function (Ricard-Blum, 2011) which is crucial for overall health and lifespan. Most importantly, in dogs affected by mitral valve disease, damage in collagen matrix, reduction of collagen in lesions and presence of immature collagens were observed in the affected tissue (Lu et al., 2015; Hadian et al., 2007; Markby et al., 2017). Changes in the expression of multiple collagen genes were also detected in the CKCS dogs affected with mitral valve disease (Markby et al., 2020; Reimann et al., 2024). These findings show that collagens play a crucial role in mitral valve disease and *COL9A1* and *COL19A1* may influence onset and progression of this disease in long-living dogs included in our study, contributing to their extended lifespan.

Another longevity-associated SNP on chromosome 12, was found in *SDHAF4* gene, which is one of the four assembly factors of Complex II helping with formation of its subunits. Dysfunction of Complex II can promote oxidative stress, contributing over time to cellular damage, aging, and disease (Goetzman et al., 2023). *SDHAF4* is also downregulated in cardiac muscle in response to pathological stress in human patients, leading to deficiency of Complex II (Wang et al., 2022).

A longevity-associated SNP in *B3GAT2* (GlcAT-S) gene, also located on chromosome 12, is one of the two glucuronyl transferases that regulate the biosynthesis of HNK-1 carbohydrate (Morita et al., 2008). This gene was not previously described in the context of longevity.

We have observed that the genotypes of longevity-associated SNPs on chromosomes 12 and 33 are linked together on each of the chromosomes. It suggests that the longevity in CKCS could be affected by large chromosomal regions. These haplotypes may have a larger effect since all the SNPs are inherited together as a set (Bickel et al., 2011).

## 5 Conclusions

Using GWAS we described 15 SNPs located in the genes: *B3GALNT1, NLRP1* like, *PARP14, IQCJ-SCHIP1, COL9A1, COL19A1, SDHAF4, B3GAT2,* and *DIRC2* that are associated with longevity in the purebred Cavalier King Charles Spaniel.

The current knowledge shows that all the genes with SNPs described in this study as associated with longevity in CKCS could directly or indirectly influence health and prolong life. We identified genes associated with common longevity-influencing factors such as cancer, immunity, DNA damage, cellular repair, and oxidative stress. We also found genes that may play a role in the tissue structure and formation. Most importantly, longevity-associated SNPs were found in genes that play a role in heart diseases, which are the major cause of death in the CKCS. We described SNPs in two collagen genes associated with longevity in the CKCS. Collagens were previously described to undergo morphological changes in the dogs affected with mitral valve disease, and changes in their expression in affected CKCS to have a direct effect on longevity

Our results show that the detected longevity-associated SNPs could also serve as potential markers of diseases. In addition, the prolongation of healthspan– disease-free period of life, would directly influence lifespan of the dogs. The proper separation of longevity markers and disease markers will be possible by further investigating these genes and their function in larger datasets and in interbreed analysis. This will allow us to assess the importance and significance of longevity-associated genes within and among breeds.

This study has some limitations considering the small number of samples included in GWAS. Sampling large numbers of long-living individuals is difficult since their occurrence in the general population of the breed is rather rare. Identification of candidate SNPs enables their verification in a larger sample size. To evaluate our current findings, monitoring of dogs included in this study should be done, along with the screening of the descendants of long-living individuals

Altogether, identification of SNPs associated with longevity could allow selective breeding of dogs carrying a longevity genotype and in future generations, the influence of longevity genotypes on the lifespan and healthspan could be compared to other factors, such as environment, lifestyle, and many more.

## Funding

This research received no external funding.

## Ethical statement

All samples were obtained non-invasively. Owners of the dogs collected and provided all samples for this study. Longevity and genetics research on dogs were approved by the Animal Ethicas Committee of the ZOO Tábor with an approval number 3/2017.

## Consent for publication

Not applicable.

## Availability of data and materials

The datasets used in this study are available upon request.

## CRediT authorship contribution statement

**Evžen Korec:** Writing – review & editing, Writing – original draft, Validation, Supervision, Project administration, Methodology. **Lenka Ungrová:** Writing – original draft, Visualization, Software, Methodology, Investigation, Formal analysis, Data curation. **Josef Kalvas:** Visualization, Conceptualization. **Jiří Hejnar:** Writing – review & editing, Validation, Supervision, Methodology, Conceptualization.

## Declaration of competing interest

The authors declare that they have no known competing financial interests or personal relationships that could have appeared to influence the work reported in this paper.

## Appendix Supplementary materials

Image, application 1
