# Supplementary material for: Identification of genes associated with longevity in dogs: 9 candidate genes described in Cavalier King Charles Spaniel
Source: Vet Anim Sci. 2024 Dec 16;27:100420. doi: 10.1016/j.vas.2024.100420 (PMC11737349; doi:10.1016/j.vas.2024.100420)
Supplement: Supplementary file 1 [file mmc1.docx]

Appendix


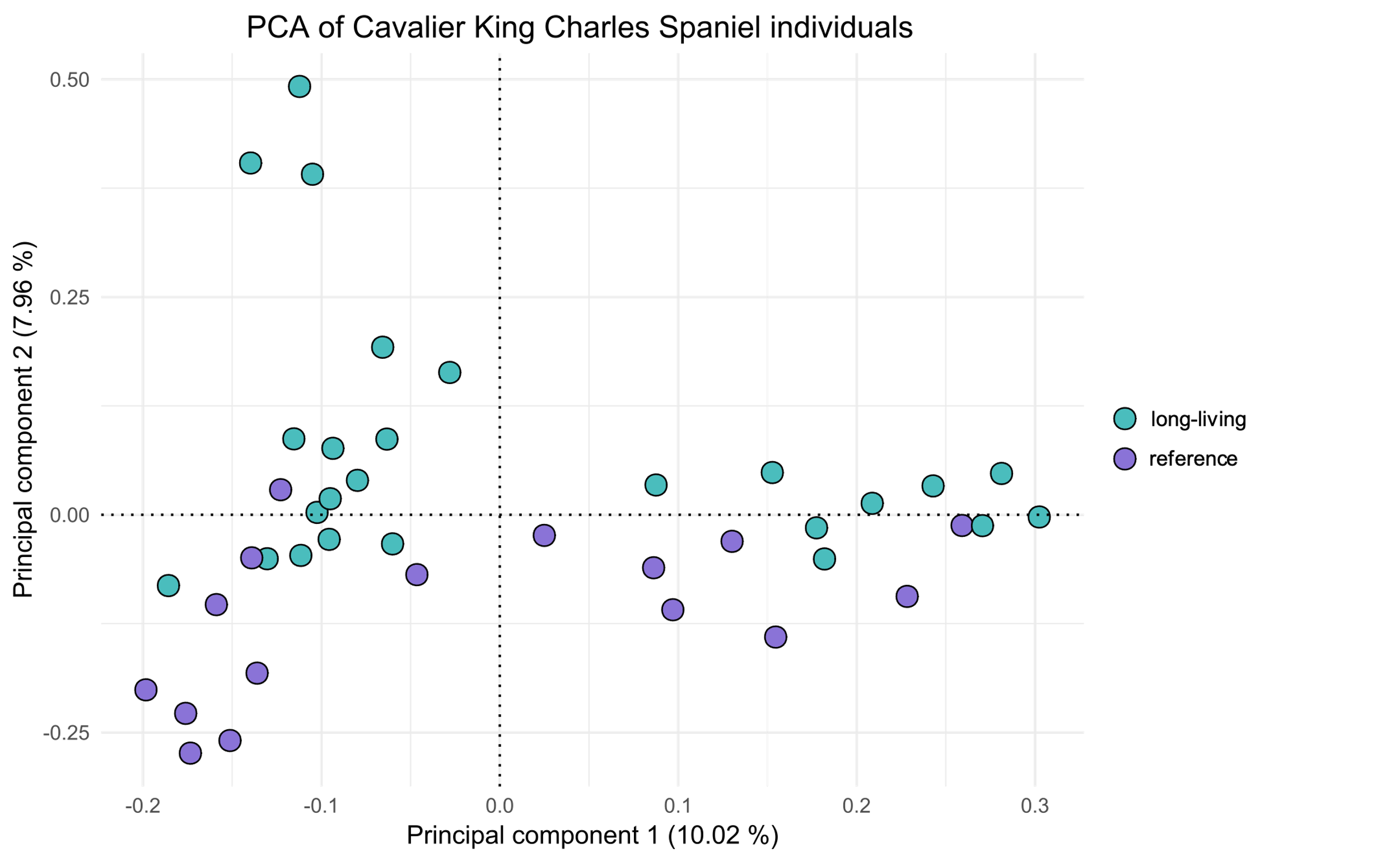


**Figure S1**. Principal component analysis of genotyped dog samples


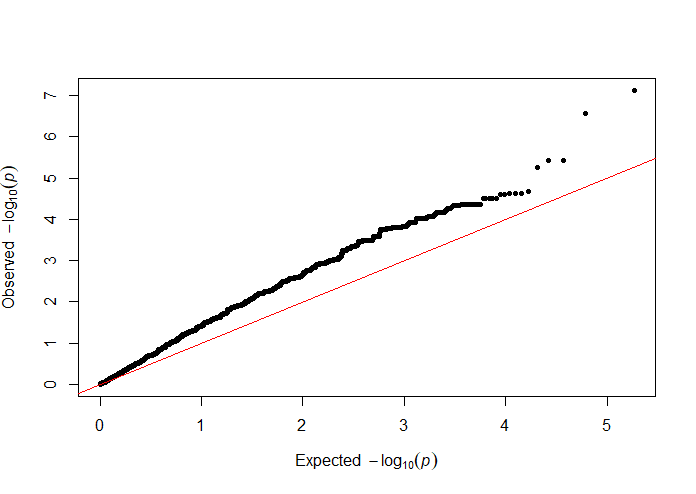


**Figure S2**. Qqplot of the GWAS results. The red line represents the expected and the black dotted line represents the observed P-values


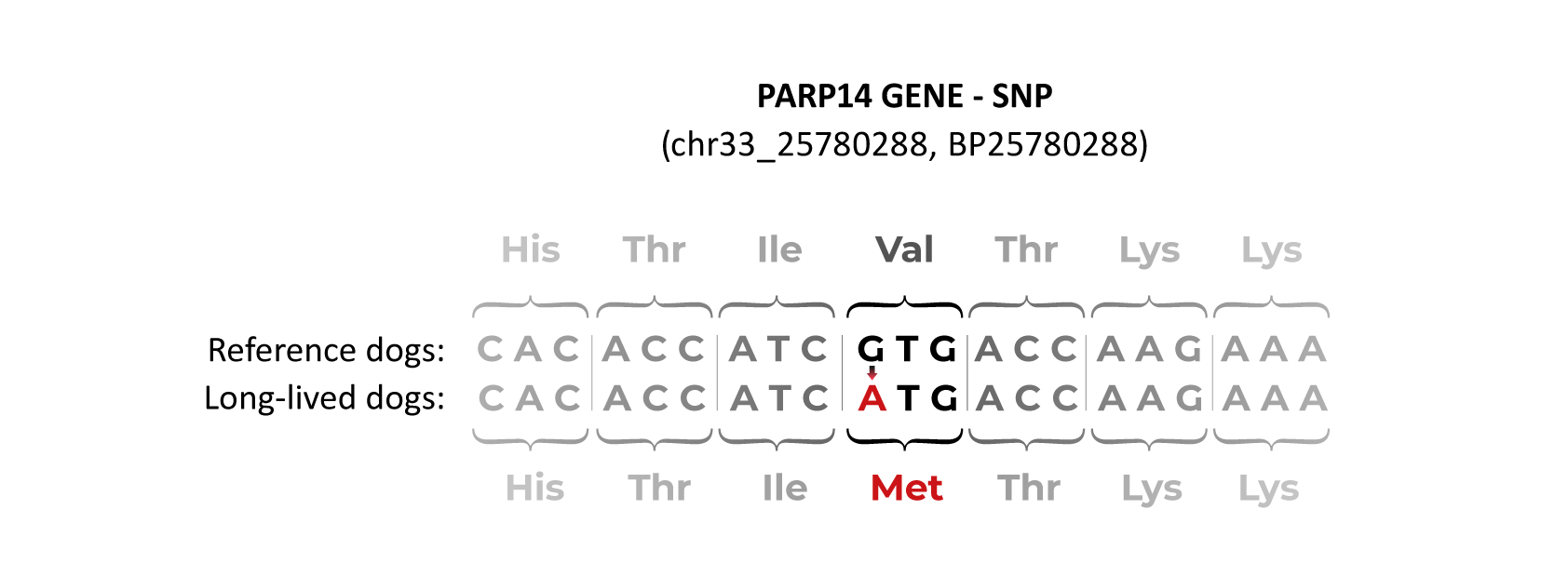


**Figure S3.** Nucleotide and amino acid change in PARP14 SNP chr33_25780288

**
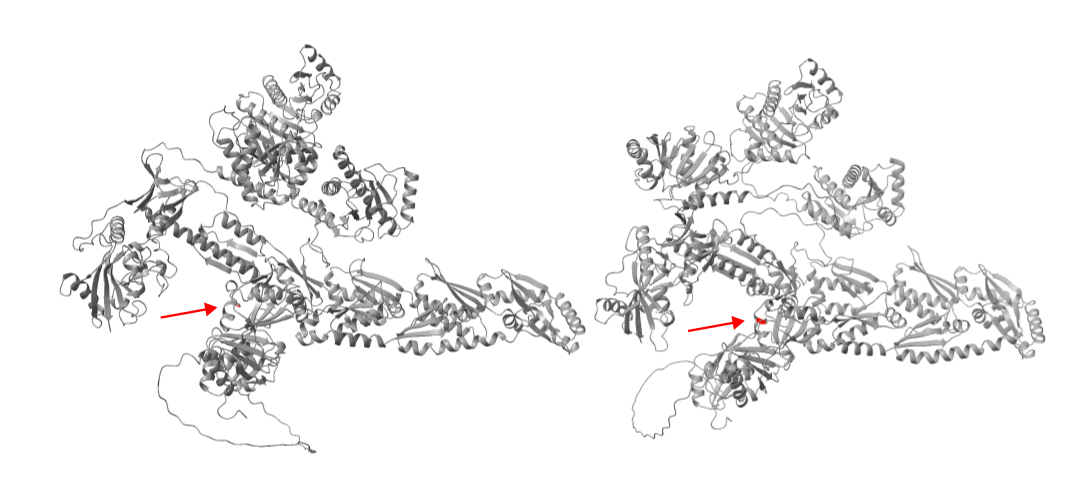
**

**Figure S4.** Prediction of the protein structure of PARP14 with amino acid change 284V->284M coloured red. The arrow indicates the position of the amino acid change.

**Table S1.** All received samples of long-lived (“old”,O) and reference (R) animals.

| **Name** | **Sex** | **Date of birth** | **Group** | **SNP genotyping** |
| --- | --- | --- | --- | --- |
| CKCFR1 | F | 29.07.2018 | R | No |
| CKCFR10 | F | 15.06.2017 | R | No |
| CKCFR11 | F | 15.06.2017 | R | Yes |
| CKCFR12 | F | 14.01.2020 | R | Yes |
| CKCFR13 | F | 06.01.2019 | R | Yes |
| CKCFR14 | F | 20.07.2016 | R | Yes |
| CKCFR23 | F | 13.08.2019 | R | Yes |
| CKCFR24 | F | 29.10.2019 | R | Yes |
| CKCFR25 | F | 20.03.2017 | R | Yes |
| CKCFR26 | F | 27.06.2018 | R | Yes |
| CKCFR27 | F | 19.05.2021 | R | No |
| CKCFR28 | F | 18.01.2017 | R | Yes |
| CKCFR29 | F | 14.07.2021 | R | Yes |
| CKCFR30 | F | 30.05.2018 | R | Yes |
| CKCFR31 | F | 12.03.2018 | R | Yes |
| CKCFR4 | F | 26.12.2020 | R | Yes |
| CKCFR5 | F | 24.03.2021 | R | Yes |
| CKCFR6 | F | 13.09.2020 | R | Yes |
| CKCFR7 | F | 18.04.2018 | R | Yes |
| CKCFR8 | F | 28.06.2020 | R | No |
| CKCFR9 | F | 08.10.2016 | R | Yes |
| CKCKMR15 | M | 14.06.2021 | R | Yes |
| CKCMR16 | M | 10.10.2018 | R | No |
| CKCMR17 | M | 15.04.2019 | R | Yes |
| CKCMR18 | M | 10.06.2018 | R | Yes |
| CKCMR19 | M | 15.09.2019 | R | Yes |
| CKCMR2 | M | 15.10.2019 | R | No |
| CKCMR20 | M | 11.09.2020 | R | No |
| CKCMR21 | M | 15.10.2019 | R | No |
| CKCMR22 | M | 20.04.2021 | R | Yes |
| CKCMR3 | M | 11.09.2020 | R | No |
| CKCFO1 | F | 14.07.2008 | O | Yes |
| CKCFO11 | F | 27.06.2009 | O | No |
| CKCFO12 | F | 02.06.2006 | O | Yes |
| CKCFO14 | F | 31.07.2009 | O | No |
| CKCFO15 | F | 27.12.2008 | O | Yes |
| CKCFO16 | F | 26.04.2009 | O | Yes |
| CKCFO17 | F | 08.05.2010 | O | Yes |
| CKCFO18 | F | 27.05.2010 | O | Yes |
| CKCFO19 | F | 20.03.2010 | O | Yes |
| CKCFO21 | F | 18.06.2010 | O | No |
| CKCFO22 | F | 10.11.2009 | O | No |
| CKCFO25 | F | 18.07.2007 | O | Yes |
| CKCFO26 | F | 26.02.2010 | O | Yes |
| CKCFO27 | F | 28.03.2010 | O | No |
| CKCFO29 | F | 14.03.2009 | O | No |
| CKCFO3 | F | 14.03.2009 | O | Yes |
| CKCFO31 | F | 28.02.2010 | O | Yes |
| CKCFO33 | F | 01.01.2010 | O | Yes |
| CKCFO34 | F | 16.04.2008 | O | Yes |
| CKCFO35 | F | 13.11.2008 | O | Yes |
| CKCFO4 | F | 16.04.2008 | O | Yes |
| CKCFO6 | F | 02.11.2007 | O | No |
| CKCFO7 | F | 27.12.2008 | O | Yes |
| CKCFO8 | F | 26.11.2009 | O | Yes |
| CKCFO9 | F | 21.04.2010 | O | No |
| CKCMO10 | M | 06.08.2009 | O | Yes |
| CKCMO13 | M | 01.11.2009 | O | Yes |
| CKCMO2 | M | 28.03.2010 | O | Yes |
| CKCMO20 | M | 27.05.2010 | O | Yes |
| CKCMO23 | M | 22.10.2009 | O | No |
| CKCMO24 | M | 28.05.2010 | O | Yes |
| CKCMO28 | M | 28.03.2010 | O | No |
| CKCMO30 | M | 23.06.2009 | O | Yes |
| CKCMO32 | M | 22.12.2009 | O | Yes |

**Table S2.** Genotypes of selected SNPs in genes of long-lived (“old”,O) and reference (R) animals. X= not successfully genotyped.

|  | **Chr** | **12** | | | | | | **33** | | | | | **34** | | | |
| --- | --- | --- | --- | --- | --- | --- | --- | --- | --- | --- | --- | --- | --- | --- | --- | --- |
|  | **Gene:** | **COL19A1** | | **COL9A1** | | **SDHAF4** | **B3GAT2** | **PARP14** | | | **DIRC2** | | **B3GALNT1** | **NLRP1** | **SDHAF4** | |
| **Name** | **Group** | **BICF2S23153539** | **TIGRP2P163151** | **chr12_32764496** | **BICF2P994617** | **BICF2P1328885** | **BICF2S2455372** | **BICF2S23418457** | **chr33_25780288** | **BICF2S2454474** | **BICF2S2361914** | **BICF2S23212476** | **BICF2G630458551** | **BICF2S23061012** | **chr34_26011266** | **TIGRP2P397333** |
| CKCFR11 | R | CC | GG | TT | TT | TT | AA | TT | AA | TT | GG | AA | AG | AG | AG | GG |
| CKCFR12 | R | CC | GG | TT | TT | TT | AA | TT | AA | TT | GG | AA | AG | AG | GG | GG |
| CKCFR13 | R | X | X | X | X | X | X | X | X | X | X | X | X | X | X | X |
| CKCFR14 | R | TC | AG | CT | CT | CT | GA | TT | AA | TT | GG | AA | AA | AA | AA | AG |
| CKCFR23 | R | CC | GG | TT | TT | TT | AA | TT | AA | TT | GG | AA | AG | AG | AG | AG |
| CKCFR24 | R | CC | GG | TT | TT | TT | AA | TT | AA | TT | GG | AA | AG | AG | AG | AG |
| CKCFR25 | R | CC | GG | TT | TT | TT | AA | GG | GG | CC | AA | GG | AG | AG | AG | GG |
| CKCFR26 | R | TC | AG | CT | CT | CT | GA | TT | AA | TT | GG | AA | AA | AA | AA | AA |
| CKCFR28 | R | CC | GG | TT | TT | TT | AA | TT | AA | TT | GG | AA | AA | AA | AA | AA |
| CKCFR29 | R | TC | AG | CT | CT | CT | GA | TT | AA | TT | GG | AA | AA | AA | AA | AA |
| CKCFR30 | R | CC | GG | TT | TT | TT | AA | TT | AA | TT | GG | AA | AA | AG | AG | AG |
| CKCFR31 | R | CC | GG | TT | TT | TT | AA | TT | AA | TT | GG | AA | AA | AA | AA | GG |
| CKCFR4 | R | CC | GG | TT | TT | TT | AA | TT | AA | TT | GG | AA | AG | AG | AA | GG |
| CKCFR5 | R | CC | GG | TT | TT | TT | AA | TT | AA | TT | GG | AA | AG | AG | AG | GG |
| CKCFR6 | R | CC | GG | TT | TT | TT | AA | TT | AA | TT | GG | AA | AA | AA | AA | AA |
| CKCFR7 | R | X | X | X | X | X | X | X | X | X | X | X | X | X | X | X |
| CKCFR9 | R | CC | GG | TT | TT | TT | AA | TT | AA | TT | GG | AA | AG | AG | AG | AG |
| CKCMR15 | R | TC | AG | CT | CT | CT | GA | TT | AA | TT | GG | AA | GG | GG | GG | GG |
| CKCMR17 | R | X | X | X | X | X | X | X | X | X | X | X | X | X | X | X |
| CKCMR18 | R | X | X | X | X | X | X | X | X | X | X | X | X | X | X | X |
| CKCMR19 | R | X | X | X | X | X | X | X | X | X | X | X | X | X | X | X |
| CKCMR22 | R | CC | GG | TT | TT | TT | AA | TT | AA | TT | GG | AA | AA | AA | AA | AG |
| CKCFO1 | O | TT | AA | CC | CC | CC | GG | GG | GG | CC | AA | GG | GG | GG | GG | GG |
| CKCFO12 | O | TT | AA | CC | CC | CC | GG | TT | AA | TT | GG | AA | GG | GG | GG | GG |
| CKCFO15 | O | TC | AG | CT | CT | CT | GA | GG | GG | CC | AA | GG | GG | GG | AG | GG |
| CKCFO16 | O | TC | AG | CT | CT | CT | GA | GT | GA | CT | AG | GA | GG | GG | GG | GG |
| CKCFO17 | O | TC | AG | CT | CT | CT | GA | TT | AA | TT | GG | AA | AG | AG | AG | GG |
| CKCFO18 | O | TT | AA | CC | CC | CC | GG | GT | GA | CT | AG | GA | GG | GG | GG | GG |
| CKCFO19 | O | TC | AG | CT | CT | CT | GA | GT | GA | CT | AG | GA | GG | GG | AG | GG |
| CKCFO25 | O | TT | AA | CC | CC | CC | GG | GT | GA | CT | GG | AA | AG | AG | AA | AG |
| CKCFO26 | O | CC | GG | TT | TT | TT | AA | GT | GA | CT | AG | GA | GG | GG | GG | GG |
| CKCFO3 | O | TC | AG | CT | CT | CT | GA | GG | GG | CC | AG | GA | GG | GG | GG | GG |
| CKCFO31 | O | TC | AG | CT | CT | CT | GA | GT | GA | CT | AG | GA | GG | GG | GG | GG |
| CKCFO33 | O | TC | AG | CT | CT | CT | GA | GT | GA | CT | AA | GG | GG | GG | GG | GG |
| CKCFO34 | O | CC | GG | TT | TT | TT | AA | GT | GA | CT | AG | GA | GG | GG | GG | GG |
| CKCFO35 | O | TT | AA | CC | CC | CC | GG | GG | GG | CC | AA | GG | GG | GG | AG | GG |
| CKCFO4 | O | TC | AG | CT | CT | CT | GA | GT | GA | CT | AG | GA | AG | AG | AG | GG |
| CKCFO7 | O | TC | AG | CT | CT | CT | GA | GG | GG | CC | AA | GG | AG | AG | AG | GG |
| CKCFO8 | O | CC | GG | TT | TT | TT | AA | TT | AA | TT | GG | AA | GG | GG | GG | GG |
| CKCMO10 | O | X | X | X | X | X | X | X | X | X | X | X | X | X | X | X |
| CKCMO13 | O | TT | AA | CC | CC | CC | GG | TT | AA | TT | GG | AA | AG | AG | AG | GG |
| CKCMO2 | O | TC | AG | CT | CT | CT | GA | GT | GA | CT | AG | GA | GG | GG | GG | GG |
| CKCMO20 | O | TT | AA | CC | CC | CC | GG | GT | GA | CT | AG | GA | GG | GG | GG | GG |
| CKCMO24 | O | X | X | X | X | X | X | X | X | X | X | X | X | X | X | X |
| CKCMO30 | O | CC | GG | TT | TT | TT | AA | GG | GG | CC | AA | GG | GG | GG | GG | GG |
| CKCMO32 | O | TC | AG | CT | CT | CT | GA | GT | GA | CT | AG | GA | GG | GG | GG | GG |
| CKCMO37 | O | CC | GG | TT | TT | TT | AA | TT | AA | TT | GG | AA | GG | GG | GG | GG |
| CKCMO5 | O | TT | AA | CC | CC | CC | GG | TT | AA | TT | GG | AA | AG | AG | AG | AG |
